# CAR‐T cell therapy for lung cancer: Potential and perspective

**DOI:** 10.1111/1759-7714.14375

**Published:** 2022-03-15

**Authors:** Long Chen, Fukun Chen, Jindan Li, Yongzhu Pu, Conghui Yang, Yue Wang, Yujie Lei, Yunchao Huang

**Affiliations:** ^1^ Department of PET/CT Center Yunnan Cancer Hospital, The Third Affiliated Hospital of Kunming Medical University, Cancer Center of Yunnan Province Kunming China; ^2^ Department of Nuclear Medicine Yunnan Cancer Hospital, The Third Affiliated Hospital of Kunming Medical University, Cancer Center of Yunnan Province Kunming China; ^3^ Department of Thoracic Surgery I Key Laboratory of Lung Cancer of Yunnan Province, Yunnan Cancer Hospital, The Third Affiliated Hospital of Kunming Medical University, Cancer Center of Yunnan Province Kunming China

**Keywords:** chimeric antigen receptor‐modified T cellsimmunotherapylung cancersolid tumortargeting specific antigens

## Abstract

Lung cancer is the highest incidence and mortality of all cancers around the world. In the present immunotherapy era, an increasing number of immunotherapeutic agents including monoclonal antibody‐targeted drugs have been used in the clinical treatment of malignancy, but it still has many limitations. Chimeric antigen receptor‐modified T (CAR‐T) cells, a novel adoptive immunotherapy strategy, have not only been used successfully against hematological tumors, but have also opened up new avenues for immunotherapy of solid tumors, including lung cancer. However, targeting lung cancer‐specific antigens using engineered CAR‐T cells is complicated by the lack of proper tumor‐specific antigens, an immunosuppressive tumor microenvironment, a low level of CAR‐T cell infiltration into tumor tissues, along with off‐target effect, etc. Simultaneously, the clinical application of CAR‐T cells remains limited because of many challenges such as tumor lysis syndrome, neurotoxicity syndrome, and cytokine release syndrome. In this review, we outline the basic structure and generation characteristic of CAR‐T cells and summarize the common tumor‐associated antigens in clinical trials of CAR‐T cell therapy for lung cancer, and point out the current challenges and new strategies, aiming to provide new ideas and approaches for the pre‐clinical experiments and clinical trials of CAR‐T cell therapy in lung cancer.

## Graphical abstract

The clinical application process of CAR‐T cell immunotherapy. The clinical process of CAR T is as listed: T lymphocyte collection; CAR T‐cell manufacturing; CAR T‐cell amplification and screening in vitro; CAR‐T cells are infused back into patients; CAR‐T cells are transported to the tumor site and perform their function in the tumor microenvironment.

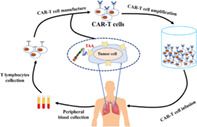

## INTRODUCTION

Lung cancer is one of the malignant tumors of the respiratory system with a highly lethal malignancy and poor prognosis.^1^, ^2^ According to Global Cancer Statistics in 2020,^3^ there will be a total of 2 206 771 new cases of lung cancer and 1 796 144 deaths, with deaths accounting for more than 80% of new cases, ranking second among causes of cancer deaths worldwide and first in China, and expected to rise to first in the United States in 2030.^4^ Meanwhile, more than 90% of lung cancer patients are diagnosed at an advanced stage that often misses the best time for treatment, and the 5‐year survival rate is only approximately 10%–20%.^5^ To date, the main treatment strategy of lung cancer is still surgical resection combined with adjuvant therapy, but only 20% of patients are suitable for surgery, and 80% of patients experience recurrence and eventually die after surgery.^6^ Therefore, it is important to seek a new treatment strategy to impede tumor progression and prolong the survival time of patients with lung cancer.

Growing evidence has illustrated that immunotherapy, especially monoclonal antibody‐targeted drugs, has been increasingly used in the clinical treatment of lung cancer, but still has many limitations.^7^ Interestingly, clinical studies have shown that chimeric antigen receptor (CAR)‐T cells served as a kind of over‐the‐top T‐cell immunotherapy, which first, isolated T‐cells from patients' blood and genetically engineered the T cells to recognize antigens on tumor cells and kill them and is considered as safe and reliable immunotherapy in malignant tumors.^8^ At present, CAR‐T cell immunotherapy has achieved great success in hematological malignancies with an overall remission rate reaching more than 80%.^9^ For example, clinical trials have verified that CAR‐T cells targeting CD19 exhibited a long‐lasting remission effect on drug‐resistant B‐cell malignancies, and the cure rate for patients with relapsed and refractory acute B‐lymphocytic leukemia was about 80%–90%.^10^ Nowadays, there are five CAR‐T products targeting CD19 in the treatment of hematological malignancies that have been approved by the Food and Drug Administration (FDA) , which brought a new direction of cancer immunotherapy and antitumor road. Simultaneously, the success of CAR‐T cell immunotherapy in the treatment of hematological malignancies provides new hope to cure solid tumors, and a range of solid tumors CAR‐T cell target antigens have been identified and are used in ongoing early clinical trials.^11^ Several scientists have focused their attention on CAR‐T cell immunotherapy for the treatment of lung cancer and made good progress in clinical trials.^12^, ^13^ The results of the above studies indicated that CAR‐T cell immunotherapy might be a novel strategy for lung cancer treatment.

The purpose of this review is to outline the recent research advances in CAR‐T cell immunotherapy for lung cancer, including the structure and generation of CAR‐T cells and antigens targeted. Moreover, we focus on the main challenges and future prospects of CAR‐T cell immunotherapy against lung cancer, aiming to provide new ideas for the clinical trial design and treatment of lung cancer immunotherapy.

## THE STRUCTURE AND GENERATION OF CAR‐T CELLS

### The structure of CAR‐T cells

**FIGURE 1:**
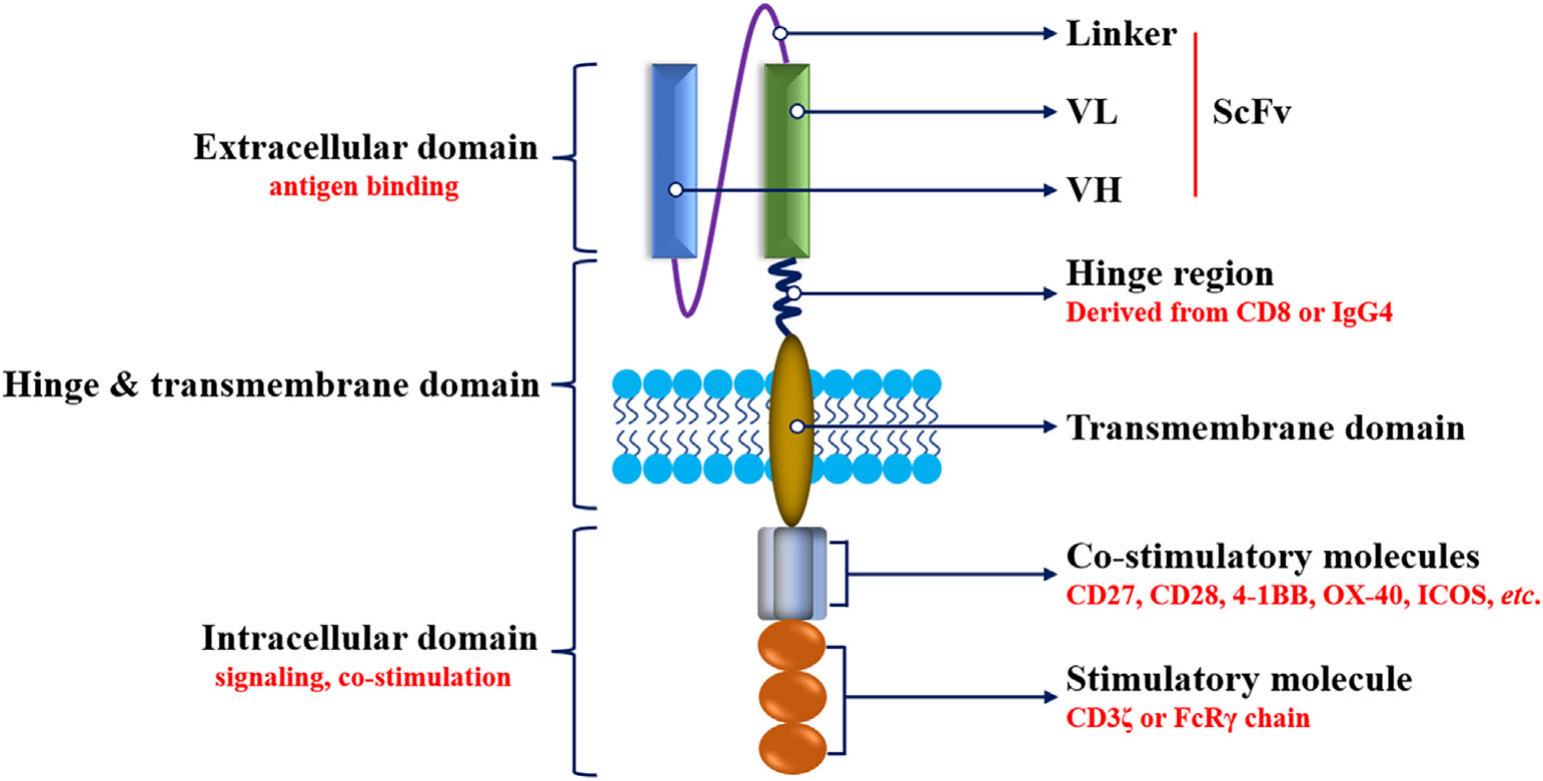
The structure of CAR. ICOS, inducible costimulatory; ScFv, single‐chain variable fragment; VH, heavy chain variable; VL, light chain variable

CAR‐T cells, as one of the emerging treatment strategies for cancer immunotherapy, have become a new hot spot and focus in the field of cancer therapy research in recent years. CAR‐T cells are genetically engineered to isolate the patient's T cells outside the body and expressed single‐chain antibodies that specifically recognize and bind to antigens (e.g., CD19) on cancer cells.^14^ CAR is mainly composed of an extracellular antigen recognition domain (ectodomain), a hinge and transmembrane domain, and an intracellular signal transduction domain (endodomain) (Figure 1). The main structure of the extracellular antigen recognition domain is the single‐chain variable fragment (scFv) of the target antigen–antibody, which consists of the heavy chain variable regions (VH) and the light chain variable regions (VL) of the specific antibody to the tumor‐associated antigen (TAA), preserving the recognition ability of the antibody, and this type of recognition ensures the specificity of the T cells in their killing action. The hinge and transmembrane structural domains are usually the transmembrane regions of CD8α or CD28, which mainly serve to connect the extracellular and intracellular structural domains and contribute to the mutual recognition of CAR and antigen and the recruitment of stimulatory signals for CAR‐T activation.^15^ The length or flexibility of the transmembrane structural domain can also affect the function of CAR.^16^ The intracellular signal transduction structural domain mainly consists of the stimulatory factor CD3ζ chain and is often combined with costimulatory molecules such as CD27, CD28, CD134 (OX‐40), and CD137 (4‐1BB), which initiate the activation of T cell function and also contribute to T cell proliferation and lifespan extension.^17^

### The generation of CAR‐T cells

**FIGURE 2:**
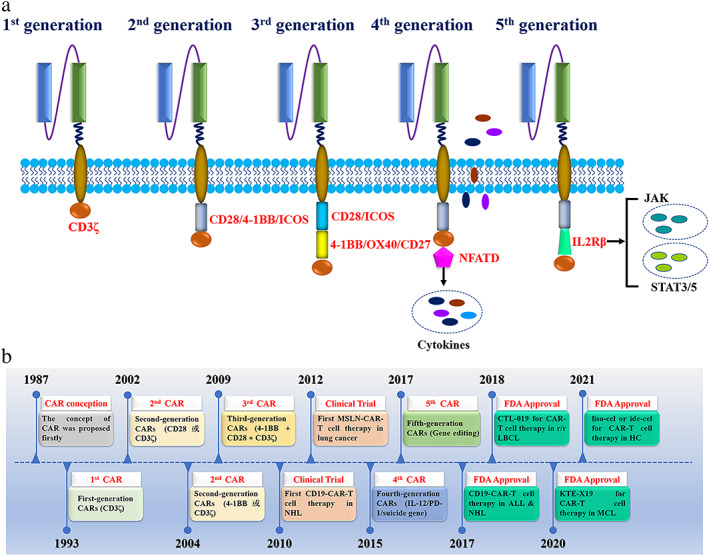
Different generations of CARs and development history. (a) The construction of 1st, 2nd, 3rd, 4th, and 5th generation CARs; (b) historic timeline of the development of CAR‐T cells. ALL, acute lymphocytic leukemia; CAR, chimeric antigen receptor; CTL‐019, tisagenlecleuel; EGFR, epidermal growth factor receptor; FDA, US Food and Drug Administration; HC, hematological cancer; ide‐cel, idecabtagene vicleucel; JAK, Janus kinase; KTE‐X19, brexucabtagene autoleucel; LBCL, large B cell lymphoma; liso‐cel, lisocabtagene maraleucel; MCL, mantle cell lymphoma; NFATD, nuclear factor of activated T cells; NHL, non‐Hodgkin lymphoma; r/r, relapsed/refractory; STAT, signal transducer and activator of transcription

To date, CAR‐T cells are classified into five generations based on their intracellular signaling structural domains, with the main differences between CAR‐T cell generations being specific co‐stimulatory molecules (Figure 2). The first generation of CAR‐T cells included only CD3ζ as an intracellular signaling endodomain that fuses with extracellular scFv to modify and activate T cells.^18^ Because first‐generation CAR‐T cells do not have co‐stimulatory molecules, they cannot provide prolonged triggering of T cell activation and therefore, have a little antitumor effect. To overcome this drawback, co‐stimulatory molecules (e.g., CD28, 4‐1BB, and inducible costimulatory [ICOS]) were added to the second‐generation CARs structure to enhance T cell proliferation viability.^19^ Previous studies have demonstrated that CD28‐CAR‐T cells were more potent in killing cancer cells, as well as 4‐1BB‐CAR‐T cells exhibited lower depletion rates and longer‐lasting killing effects on cancer cells.^20^ To improve the tumor killing ability of T cells, the third‐generation CARs are based on the second generation by continuing to add co‐stimulatory molecules to CARs, such as CD28 combined with OX‐40/4‐1BB.^21^ Several studies showed that the levels of cytokine secretion in the third generation CAR‐T cells were upregulated and the inhibition effect on cancer cell proliferation was enhanced.^22^, ^23^ The fourth‐generation CARs, also known as T cells redirected for universal cytokine‐mediated killing (TRUCKs),^24^ have increased genes encoding cytokines to further activate and recruit more immune cells against the immunosuppressive local tumor microenvironment by secreting a large number of inflammatory cytokines, such as interleukin (IL)‐12, IL‐15, and granulocyte‐macrophage colony‐stimulating factor, which contributes to the immunotherapy of solid tumors. Recent studies have pointed out that fifth‐generation CARs contain a segment of IL‐2 receptor β (IL‐2Rβ) instead of OX‐40/CD27. The IL‐2Rβ segment facilitates the levels of Janus kinases and signal transducers and activators of transcription‐3/5 in tumors,^25^, ^26^ but the safety and efficacy of fifth‐generation CARs are under investigation. Notably, the construction of complex overexpression fragments may affect the transduction efficiency of CAR‐T cells and even accelerate the depletion of CAR‐T cells.^27^ To date, the second‐generation CAR‐T is more widely used and has more stable effects.

## PROMISING TARGET ANTIGENS FOR CAR‐T CELL THERAPY IN CLINICAL TRIALS OF TREATMENT FOR LUNG CANCER

The first step in successful adoptive T cell therapy is to select the optimal TAA for CAR‐T cells. Of note, the ideal TAA is expressed only in tumor cells and not absent in normal tissue cells (or, if expressed, at very low levels),^28^, ^29^ which is the most ideal antigenic target for CAR‐T cell therapy. However, it is difficult to obtain the ideal TAA for CAR‐T cell therapy in solid tumors as CD19 in hematological cancers.^30^, ^31^ Based on the previous clinical trials, we summarized a list of TAAs as a target of CAR‐T cells in patients with lung cancer (Table 1).

**TABLE 1:** Targeting antigens of lung cancer for CAR‐T cell therapy registered in ClinicalTrials.gov

Targeted antigen	Estimated enrollment	Phase	Age (y)	Status	First posted	Sponsor	ClinicalTrial ID
CEA	40	I/II	18–75	Recruiting	Apr 16, 2020	Chongqing Precision Biotech, China	NCT04348643
CEA	75	I	18–80	Unknown	Jan 29, 2015	Southwest Hospital, China	NCT02349724
CD276	24	Early I	1–70	Not yet recruiting	Apr 29, 2021	PersonGen BioTherapeutics (Suzhou), China	NCT04864821
EGFR	11	I	18–75	Recruiting	Nov 6, 2019	Sun Yat‐sen University, China	NCT05060796
EGFR	11	Early I	18–75	Recruiting	Sep 29, 2021	Second Affiliated Hospital of Guangzhou Medical University, China	NCT05060796
HER2	45	I	≥18	Recruiting	Nov 14, 2018	Baylor College of Medicine, USA	NCT03740256
HER2	18	I	≥18	Recruiting	Dec 9, 2020	Carisma Therapeutics, USA	NCT04660929
HER2	10	I/II	18–80	Unknown	Sep 5, 2013	Chinese PLA General Hospital, China	NCT01935843
MSLN	15	I/II	18–70	Terminated	Apr 24, 2012	National Cancer Institute, USA	NCT01583686
MSLN	27	I	≥18	Recruiting	Feb 15, 2017	University of Pennsylvania, USA	NCT03054298
MUC1	20	I/II	18–70	Unknown	Oct 27, 2015	PersonGen BioTherapeutics (Suzhou), China	NCT02587689
MUC1	60	I/II	18–70	Recruiting	May 16, 2018	First Affiliated Hospital of Guangdong Pharmaceutical University, China	NCT03525782
PD‐L1	20	I/II	18–65	Unknown	Aug 10, 2016	Shanghai International Medical Center, China	NCT02862028
ROR1	60	I	≥18	Recruiting	Mar 11, 2016	Fred Hutchinson Cancer Research Center, USA	NCT02706392
TnMUC1	112	I	≥18	Recruiting	Jul 18, 2019	Tmunity Therapeutics, USA	NCT04025216
PD‐L1 and CD80/CD86	10	Early I	≥18	Unknown	Feb 23, 2017	Second Xiangya Hospital of Central South University, China	NCT03060343
GPC3 or TGFβ	30	I	18–75	Recruiting	Jun 26, 2017	Second Affiliated Hospital of Guangzhou Medical University, China	NCT03198546
αPD1 and MSLN	10	Early I	18–70	Recruiting	Jul 28, 2020	Wuhan Union Hospital, China	NCT04489862
NY‐ESO‐1 or EGFR V III	73	I/II	4–70	Recruiting	Aug 20, 2018	Shenzhen BinDeBio, China	NCT03638206
MAGE‐A1, MAGE‐A4, MucI, GD2, and MSLN	20	I/II	18–80	Recruiting	Nov 29, 2017	Shenzhen Geno‐Immune Medical Institute, China	NCT03356808
HER2, MSLN, PSCA, MUC1, GPC3, Lewis‐Y, AXL, EGFR, or B7‐H3	30	I	18–75	Recruiting	June 23, 2017	The Second Affiliated Hospital of Guangzhou Medical University, China	NCT03198052
HER2, MSLN, PSCA, MUC1, Lewis‐Y, GPC3, AXL, EGFR, Claudin18.2/6, ROR1, GD1, or B7‐H3	40	I	18–85	Recruiting	Apr 13, 2021	Second Affiliated Hospital of Guangzhou Medical University, China	NCT04842812

Abbreviations: CEA, carcinoembryonic antigen; EGFR, epidermal growth factor receptor; GPC3, glypican‐3; HER2, human epidermal growth factor receptor 2; MSLN, mesothelin; MUC1, mucin 1; PD‐L1, programmed death‐ligand 1; PSCA, prostate stem cell antigen; ROR1, inactive tyrosine‐protein kinase transmembrane receptor.

### Epidermal growth factor receptor

Numerous studies have confirmed that epidermal growth factor receptor (EGFR) belongs to the ErbB family of growth factor receptor tyrosine kinases,^32^ that are highly expressed on the membrane surface of many solid tumor cells and are associated with tumor angiogenesis, metastasis, and recurrence.^33^ Scharpenseel et al.^34^ demonstrated that EGFR expression was upregulated in the tissues of patients with non–small cell lung cancer (NSCLC) (primary tumors) and brain metastasis. Moreover, EGFR may serve as an effective target for the diagnosis and treatment of solid tumors.^35^, ^36^ Importantly, EGFR has been served as a novel target for antibody‐based immunotherapy in multiple tumors, including lung cancer.^37^ The results of an EGFR‐positive relapsed/refractory NSCLC clinical trial (NCT01869166) showed that none of the patients got significant toxic side effects after anti‐EGFR CAR‐T cell therapy, two patients achieved partial remission (PR) and five patients had stable disease (SD) for 2–8 months. Pathological biopsies revealed that CAR‐T cells targeting EGFR could infiltrate tumor tissues and induce EGFR‐specific cytotoxicity. In addition, Zhang et al.^38^ demonstrated that EGFR variant III (EGFRvIII)‐CAR‐T cells could effectively identify and kill EGFRvIII‐positive lung cancer cells by releasing cytokines including interferon γ (IFN‐γ) and tumor necrosis factor α (TNF‐α), as well as contributing to inhibiting the growth of transplanted tumors in vivo, indicating that CAR‐T cells targeting EGFRvIII are an effective therapeutic strategy to prevent recurrence and metastasis of lung cancer after surgery. Another phase I trial (NCT04153799) confirmed that C‐X‐C chemokine receptor (CXCR) type 5‐modified anti‐EGFR CAR‐T cells were used to evaluate the safety and feasibility in EGFR‐positive NSCLC patients. The above studies suggested that EGFR‐CAR‐T cells might function as immune killers through modulating tumor immune microenvironment in lung cancer.

### Mesothelin

Mesothelin (MSLN) is a cell adhesion glycoprotein that promotes cancer invasion and metastasis.^39^, ^40^ MSLN overexpression was positively correlated with high tumor aggressiveness and poor prognosis in lung cancer patients,^41^ as well as acted as a diagnostic and therapeutic target in malignant pleural mesothelioma and lung cancer.^42^ MSLN has been reported to be a more desirable TAA for CAR‐T therapy in solid tumors.^43^, ^44^, ^45^ In addition, multiple clinical studies of anti‐MSLN CAR‐T cells for the treatment of lung cancer are underway (NCT02414269 and NCT02580747). For example, an earlier clinical trial (NCT01583686) at the National Institutes of Health (NIH) was conducted to test the safety and feasibility of MSLN‐CAR‐T cells to treat advanced malignant tumors (including lung, pancreatic, malignant mesothelioma, cervical, and ovarian cancers). The results showed that a total of enrolling 15 patients in phase I clinical trial had the incidence of serious adverse reactions was 40% (6/15), including one case each of anemia, thrombocytopenia, constipation, and hypoxemia, and two cases of lymphopenia, whereas only one patient had stable disease (SD) during the 3.5‐month observation period. In vivo experiments showed that MLSN‐CAR‐T cells inhibited xenografts growth by exhibiting a significantly higher ability to kill cancer cells than T cells in NSCLC.^46^ Another study confirmed that MSLN‐CAR‐T cell therapy significantly hampered tumor growth via thoracic injection and persisted in mice bodies for a long time.^47^ Furthermore, the dual CAR‐T cells targeting MSLN and carcinoembryonic antigen (CEA) antigens have stronger anti‐pancreatic cancer activity.^48^ Taken together, MSLN may be an effective TAA for CAR‐T cells in solid tumor immunotherapy.

### Mucin 1

Mucin 1 (MUC1) is a transmembrane protein that facilitates cancer cell adhesion and metastasis.^49^, ^50^ Previous studies have confirmed that the expression of MUC1 was significantly higher in lung cancer tissues than in normal lung tissues.^51^ Another study showed that knockdown of MUC1 significantly suppressed lung cancer cell proliferation and induced apoptosis in vitro, as well as inhibited tumor growth and metastasis in an orthotopic mouse model of lung cancer.^52^ Of note, MUC1 has been reported as a reliable target for immunotherapy of small cell lung cancer.^53^, ^54^ Wei et al.^55^ showed that CAR‐T cells targeting prostate stem cell antigen (PSCA) and MUC1 significantly eliminated tumor cells of combination PSCA and MUC1 positive in NSCLC. In addition, there are ongoing anti‐MUC1 CAR‐T cells successively used in clinical trials (NCT02587689, NCT03525782, NCT03356808, NCT03198052, and NCT04842812) for lung cancer. The above studies indicate that anti‐MUC1 CAR‐T cells may be an effective strategy for immunotherapy of patients with lung cancer.

### Inactive tyrosine‐protein kinase transmembrane receptor

Inactive tyrosine‐protein kinase transmembrane receptor (ROR1), a tyrosine kinase‐like orphan receptor, is upregulated in B cell chronic lymphoblastic leukemia, mantle cell lymphoma, acute lymphocytic leukemia, lung cancer, breast cancer, pancreatic cancer, and ovarian cancer, but has very low expression in normal tissues.^56^ A clinical trial at the Fred Hutchinson Cancer Research Center (NCT02706392) evaluated anti‐ROR1 CAR‐T cells for the treatment of advanced ROR1‐positive and stage IV NSCLC and triple‐negative breast cancer (TNBC), and the results showed that at least 6 of 30 patients recruited did not show dose‐limiting toxicity. Wallstabe et al.^57^ demonstrated that treatment with anti‐ROR1 CAR‐T cells can effectively kill NSCLC and TNBC cells by a three‐dimensional tumor model. Therefore, CAR‐T cells targeting ROR1 provide a new strategy for the clinical treatment of lung cancer.

### Carcinoembryonic antigen

CEA is highly expressed in lung cancer,^58^ but lowly expressed in normal tissues and differentiated cells. Analysis of the The Cancer Genome Atlas (TCGA) database revealed that lung cancer patients with high CEA expression had a poor prognosis. Preclinical data have confirmed that the serum concentrations of CEA in patients with advanced NSCLC were correlated with the occurrence of brain metastases^59^ and high CEA expression associated with clinicopathological characteristics in lung cancer patients including lymph node metastasis and vascular infiltration.^60^ The above results provide a theoretical basis for clinical trials to assess the safety, efficacy, and maximum tolerated dose of anti‐CEA CAR‐T cell therapy in CEA‐positive lung cancer (NCT02349724 and NCT04348643).

### Human epidermal growth factor receptor 2

Previous studies have confirmed that human epidermal growth factor receptor 2 (HER2) was highly expressed in lung cancer^61^ and facilitated the proliferation, invasion, and angiogenesis of cancer cells.^62^ HER2 served as a promising biomarker for the diagnosis and treatment of lung cancer.^63^, ^64^ Zhao et al.^65^ reported that anti‐HER2 CAR‐T cells exhibited an antitumor effect on HER2‐positive tumors in a xenogeneic mouse model. In addition, many scholars from both domestic and international are conducting clinical trials of anti‐HER2 CAR‐T cells for lung cancer (NCT03198052, NCT01935843, NCT02713984, NCT03740256, and NCT04660929) to validate the safety and efficacy of HER2‐CAR‐T cells in the treatment of HER2‐positive lung cancer. To date, clinical outcomes of anti‐HER2 CAR‐T cells for the treatment of lung cancer have not been reported.

### Programmed death‐ligand 1

The therapeutic effect of CAR‐T cell therapy for NSCLC was associated with an immunosuppressive tumor microenvironment.^66^ Programmed death‐ligand 1 (PD‐L1), an important immune checkpoint, was upregulated in multiple tumors,^67^ and it can inhibit T cell proliferation and activation by binding to PD‐1 on T cells, ultimately leading to immune escape of tumor cells.^68^ Previous studies have shown that PD‐L1 antibody exhibited safe and exciting results in in vitro, in vivo experiments, and clinical trials in tumors, including lung cancer.^69^, ^70^ Moreover, CAR‐T cell targeting PD‐L1 and zeushield cytotoxic T lymphocytes is being evaluated for safety and efficacy in an ongoing early‐phase I trial for relapsed or refractory NSCLC (NCT03060343). Liu et al.^71^ demonstrated that anti‐PD‐L1 CAR‐T cells significantly suppressed PD‐L1^high^ NSCLC cell proliferation and the growth of xenograft tumors in mice, as well as radiotherapy combined with anti‐PD‐L1 CAR‐T cells exhibited cytotoxic activity against PD‐L1^low^ NSCLC cells and xenograft tumors. However, a pilot study of anti‐PD‐L1 CAR‐T cell immunotherapy for advanced lung cancer in a phase I trial was terminated because of serious adverse events (NCT03330834). Intriguingly, anti‐MUC1 CAR‐T combining PD‐1 knockdown cells were used to assess the safety and efficacy for patients with advanced NSCLC in a phase II trial (NCT03525782).

### B7‐H3

B7‐H3 is a member of the B7 immunoglobulin superfamily, which is highly expressed in various solid tumors, and it has received widespread attention as an effective biomarker for cancer immunotherapy.^72^ Preclinical studies have shown that B7‐H3 inhibited T cell activation and effectively suppressed the proliferation, cytokine production, and cytotoxic functions of activated T cells.^73^, ^74^ Importantly, B7‐H3 was overexpressed in tissues of patients with NSCLC,^75^ and antibody immunotherapy targeting B7‐H3 does not produce toxicity to vital organs, which makes it an ideal anticancer target. Currently, the antibody‐drug MGC018 targeting B7‐H3 has shown potent antitumor activity in patient‐derived xenograft models of breast, ovarian, and lung cancer.^76^ In addition, B7‐H3‐CAR‐T cells exhibited effective antitumor activity in ovarian cancer, neuroblastoma, and melanoma.^77^, ^78^ Tang et al.^79^ demonstrated that injection of B7‐H3‐CAR‐T cells significantly inhibited tumor growth and prolonged survival time in melanoma transplanted model mice. Similarly, several clinical trials have been designed to test the safety, tolerability, and feasibility of B7‐H3‐targeted CAR‐T cells against lung cancer such as NCT04864821 and NCT03198052.

### Other targeted antigens

Given the promise of CAR‐T cell immunotherapy in preclinical models of lung cancer, new approaches to identify additional effective antigenic targets for lung cancer are being investigated. For example, CXC chemokine receptor 4 (CXCR4) was upregulated in lung cancer tissues and cell lines,^80^ as well as served as an effective therapeutic target for NSCLC.^81^ Mao et al.^82^ found a lung adenocarcinoma‐associated MAGE‐A1 antigen via analyzing cancer/testis antigen database, and MAGE‐A1‐specific CAR‐T cell immunotherapy for lung adenocarcinoma is effective and safe. In addition, there are many other candidate antigenic targets for CAR‐T cell immunotherapy in lung cancer, including PSCA, tyrosine kinase receptor EphA2, phosphatidylinositol proteoglycan 3 (GPC3), folate receptors (FRα and FRβ), CD44v6, Lewis‐Y antigen, IL‐13Rα2, L1 cell adhesion molecule (L1CAM), and disialoganglioside (GD2),^83^, ^84^, ^85^, ^86^ which have not yet completed clinical trials.

## LIMITATIONS AND STRATEGIES OF CAR‐T CELL THERAPY IN LUNG CANCER

In general, the study on CAR‐T cell therapy for lung cancer is still in the early exploration stage. A large number of clinical trials progressed slowly and got very limited efficacy, as well as several technical bottlenecks need to be solved. Unlike hematologic malignancies, several major challenges of CAR‐T cell therapy for lung cancer include on‐target/off‐tumor toxicity, TAA heterogeneity, immunosuppressive tumor microenvironment (TME), neurological toxicity, cytokine release syndrome, etc. How to overcome these challenges is the current hot field of CAR‐T cell therapy in lung cancer.

### On‐target/off‐tumor toxicity and TAA heterogeneity

The most important problem with CAR‐T cell therapy for solid tumors is the lack of an ideal antigenic target. Numerous studies have shown that the key component to the success of these candidate target antigens of CAR‐T cells will depend on their safety and the degree of on‐target/off‐tumor toxicity.^13^ For instance, Morgan et al.^87^ reported that injection with anti‐ERBB2 CAR‐T cells resulted in a colon cancer patient with low ERBB2 expression in the normal lung tissues developing respiratory distress after 15 minutes and eventually died after 5 days. Off‐tumor toxicity of CAR‐T cells may cause normal organ dysfunction, including pulmonary fibrosis, liver damage, and gastrointestinal disorders.^88^ To avoid these risks, several strategies have been used to deal with the problem of off‐tumor toxicity and TAA heterogeneity, including targeting mutated tumor‐specific antigens, targeting multiple tumor target antigens,^89^ dual CAR system,^90^ and suicide genes,^91^ etc. Moreover, new technology single‐cell RNA sequencing may provide a more accurate target antigen expression profile for TAA selection, which can better predict the efficacy and toxicity of novel CAR‐T cell therapy in tumors.^92^ Furthermore, designing CAR‐T cells targeting multi‐targets in combination may also be an effective strategy to enhance tumor eradication.^93^ For example, Roybal et al.^94^ found that the anti‐GFP and anti‐CD19 dual‐specific CAR‐T cells significantly inhibited K562 cell proliferation and xenograft tumor growth. Preclinical studies showed that GD2‐B7H3^95^ or ROR1‐B7H3^96^ synNotch CAR‐T cells significantly killed tumor cells with high specificity and efficacy and without toxicity to normal cells expressing the target antigen.

### Immunosuppressive tumor microenvironment

The clinical efficacy of CAR‐T cell therapy for solid tumors is restricted by the immunosuppressive TME, such as hypoxia, immunosuppressive signaling by cellular immune checkpoint receptors, oxidative stress, and tumor‐derived cytokine suppression, etc.^97^, ^98^ Meanwhile, previous studies have demonstrated that tumor cells can release a variety of immunosuppressive factors, including VEGF, IL‐4, IL‐10, TGF‐β, and prostaglandin E2, leading to the activation of suppressive immune cells such as regulatory T cells, myeloid‐derived suppressor cells, and tumor‐associated macrophages.^99^ Preclinical studies found that TME has been extensively characterized as hostile for T cells.^100^, ^101^ The above studies suggest that altering the immunosuppressive effects on the TME or reconstituting the TME may enhance the anticancer effects of CAR‐T cells in solid tumors. Rafiq et al.^102^ demonstrated that PD‐1‐blocking scFv secreting CAR‐T cells significantly prolonged the survival time of transplanted tumor‐bearing mice and initiated antitumor immune responses again on tumor recurrence in mice. Chen et al.^103^ reported that CAR‐T cells overexpressing PD‐1 dominant negative receptor could act as a “decoy receptor” to bind and block PD‐L1/2 inhibitory signals. Zhou et al.^104^ reported that IL‐7/IL‐5 exhibited antitumor activity by promoting CAR‐T cell proliferation ability, reducing CAR‐T cell apoptosis, and ameliorating immunosuppressive TME. Therefore, CAR‐T cells co‐expressing immune‐related factors may be an effective strategy for the clinical treatment of lung cancer.

### Cytokine release syndrome

Cytokine release syndrome (CRS) is a systemic inflammatory response triggered by T‐cell activation, usually manifested by fever, chills, muscle pain, generalized weakness, and systemic organ failure.^105^ Clinical studies have shown that CRS was mainly caused by the activated CAR‐T cell resulted in a significant increase in the secretion of pro‐inflammatory factors (e.g., IL‐6, IFN‐γ, and TNF‐α) by immune cells (T cells, B cells, natural killer cells, and monocytes/macrophages) that disrupt the balance between pro‐inflammatory and anti‐inflammatory responses.^106^ Previous studies have proved that a controlled gene “device” for CAR‐T cells was effective in reducing pro‐inflammatory cytokines secretion and clearing CAR‐T cells from the body in time for acute toxicity.^107^, ^108^ For example, herpes simplex virus thymidine kinase (HSV‐TK), human inducible caspase 9 (iCasp9), mutant human thymidylate kinase (mTMPK), and human CD20 can be expressed in donor T cells^109^, ^110^ and have been shown to kill transduced CAR‐T cells during adverse events in the early clinical trials of immunotherapy. Similarly, Mestermann et al.^111^ found that dasatinib acted as a CAR‐T cell “switch” to control the biological function of CAR‐T cells on entry into the body and protect model mice from CRS. In addition, previous studies have demonstrated that regulating the in vivo lifespan and kinetics of CAR‐T cells by optimizing CAR gene transfection^112^ and using nanoparticles^113^ can reduce and avoid CRS. Therefore, avoiding CRS damage after CAR‐T cell immunotherapy will be a key issue to address and focus on in the treatment of lung cancer in the future.

### Neurological toxicity

Neurotoxicity, also known as CAR‐T cell‐related encephalopathy syndrome (CRES), is characterized by various neurological symptoms such as headache, aphasia, and delirium, even cerebral hemorrhage, seizures, and death.^114^ Previous studies have found that the systemic inflammatory response associated with CRS may contribute to the risk of complications CRES.^115^, ^116^ In addition, clinical studies have shown that the activation of endothelial cells facilitated the disruption of the blood–brain barrier, allowing immune effector cells and inflammatory mediators to infiltrate into the central nervous system, leading to neurotoxicity.^117^, ^118^ Simultaneously, the autopsy report of the brains with fatal CRES showed that the patient's brain had endothelial dysfunction and blood–brain barrier disruption.^119^ CRES can be largely reversible and completely resolved after treatment with tocilizumab and dexamethasone, although neurotoxicity recovery was slower after treatment with tocilizumab for CRES patients with endothelial cell activation.^117^ Collectively, an in‐depth understanding of the pathophysiology will be an important factor in reducing systemic CRES in the prospect of CAR‐T cell immunotherapy for solid tumors.

## FUTURE PERSPECTIVES FOR CAR‐T CELL THERAPY IN LUNG CANCER

The success of CAR‐T cell therapy in hematologic malignancies has brought new hope for the clinical treatment of lung cancer and has entered a phase of rapid development.^26^ However, because of the heterogeneity of malignant solid tumors and the limitations of preclinical experiments, the clinical applications of CAR‐T cells should take a more cautious approach, and future studies on CAR‐T cells may include (1) finding more stably expressed and specific target antigens; (2) modifying the structure of CAR to enhance the efficacy, specificity, and survival time of CAR‐T cells; (3) reducing the toxicity of CAR‐T cells; (4) optimizing CAR‐T cells that target the TME of lung cancer; (5) exploring combination therapies, such as combining immune checkpoint inhibitors, PD‐1 inhibitors, or dual‐CAR‐T; and (6) establishing natural ligand‐receptor‐based CAR‐T cells. These modified CARs are being studied in animal models and clinical trials in an attempt to mitigate tumor antigen heterogeneity and may eventually form the next generation of CAR‐T cells.^120^ In conclusion, the above efforts will provide safer and more effective clinical applications of CAR‐T cell immunotherapy for solid tumors such as lung cancer.

## CONCLUSION

In this review, we summarized the structure and generation of CAR‐T cells, and the most commonly TAAs used in preclinical and clinical trials of CAR‐T cell therapy against lung cancer, as well as pointed out current challenges and novel strategies. Currently, although research on CAR‐T cell immunotherapy against solid tumors is still in its infancy, the beneficial results of preliminary trials provide a theoretical foundation for their application in the subsequent clinical treatment of solid tumors. With the growing understanding of tumorigenesis, it is more important to cultivate and use patients' resources to fight the disease than other drugs. At the same time, combined with the insights into TME, screening of novel target antigens, and advances in molecular biotechnology, CAR‐T cell therapy may improve its current status in the near future. In recent years, CAR‐T cells have made great progress in the field of hematological tumors, which has stimulated the interest of many investigators to study the application of CAR‐T cells in lung cancer treatment, and several basic and early clinical trials of CAR‐T cells for solid tumors are underway. Importantly, improving the killing effect of CAR‐T cells on tumor cells and prolonging the survival time of patients with cancer are also urgent issues to be addressed in future research. In addition, combining CAR‐T cell with radio‐/chemotherapy may improve its current antitumor activity. In conclusion, with the continuous innovation of CAR‐T design ideas and treatment protocols, CAR‐T cell immunotherapy is expected to become a major “tool” for lung cancer treatment.

## AUTHOR ROLES

Long Chen and Fukun Chen designed the study and wrote this manuscript; Jindan Li, Yongzho Pu, Conghui Yang and Yue Wang compiled and analyzed the literature. Yujie Lei and Yunchao Huang proposed the study, revised and re‐organized the manuscript. All authors read and approved the final manuscript.

## DECLARATION OF CONFLICTING INTERESTS

The author(s) declared no potential conflicts of interest with respect to the research, authorship, and/or publication of this article.
